# Congenital prepubic sinus presenting with purulent discharge after circumcision: a case report

**DOI:** 10.1186/s13256-019-2019-6

**Published:** 2019-02-28

**Authors:** Yavuz Güler, Akif Erbin, Burak Üçpınar, Ömer Vural, Zeynep Tatar

**Affiliations:** 1 Department of Urology, Safa Hospital, Istanbul, TR Turkey; 2 Department of Urology, Haseki Traning and Research Hospital, Istanbul, TR Turkey; 3 Department of Pediatric Surgery, Safa Hospital, Istanbul, TR Turkey; 4 Department of Pathology, Patomer Pathological Cytological Research Center, Istanbul, TR Turkey

**Keywords:** Circumcision, Prepubic sinus, Purulent discharge, Dorsal venous thrombosis, Mondor disease

## Abstract

**Background:**

Congenital prepubic sinus is a rare anomaly found in the midline of the lower abdomen. Congenital prepubic sinus is usually asymptomatic in neonates, and a diagnosis is often achieved later in life after spontaneous extrusion of purulent material from the pre-existing hole in the pubic region. We present a case of congenital prepubic sinus presenting with purulent discharge after circumcision.

**Case summary:**

A 4-year-old Caucasian boy presented to our urology out-patient clinic with purulent discharge from the distal part of the dorsum of his penis. He had a history of circumcision performed at a different center, 6 months ago. His parents stated that although various antibiotics were used, the purulent discharge continued for 6 months and the child had no complaints before circumcision. His condition was reported as superficial dorsal venous thrombosis, known as penile Mondor disease, in magnetic resonance imaging that was performed in the previous hospital. A physical examination revealed a small pinhole lesion at the distal part of his penis and a rigid cylindrical tube extending to the proximal side of his penis. We performed fistulography by injecting contrast material through a small angiocatheter and confirmed the diagnosis of prepubic sinus. Surgical exploration was performed and a long sinus, apparently ending as a fibrous tract at the anterior surface of his pubic symphysis, was found and resected.

**Conclusions:**

Before congenital prepubic sinus surgery, it is critically important to rule out penile Mondor disease and the possibility of a circumcision complication (especially infective complications) mimicking congenital prepubic sinus.

## Background

Congenital prepubic sinus (CPS) is a rare anomaly found in the midline of the lower abdomen. It was first described by Campbell *et al*. as a duplicated dorsal urethra in 1987 [1]. The etiology is uncertain and the anatomical features often differ from each other. In most cases, the chief complaint of CPS is discharge from the sinus opening. In this case presentation, we aimed to describe the diagnosis and management of a case of CPS presenting with purulent discharge after circumcision.

## Case presentation

A 4-year-old Caucasian boy presented to our urology out-patient clinic with purulent discharge from the distal part of the dorsum of his penis. His medical and social histories were unremarkable. The child was potty-trained and his developmental milestones and psychosocial status were compliant with his percentile. There was no consanguinity between the parents and they had no inherited disease. The mother’s pregnancy period was uneventful.

Our patient had undergone circumcision at a different hospital 6 months ago. His parents stated that although various antibiotics were used, the purulent discharge had been continuing for 6 months and the child had no complaints before circumcision. On admission, his temperature was 36.4 °C, pulse was 98 beats/minute, and blood pressure was 80/50 mmHg. His condition was reported as superficial dorsal venous thrombosis, known as Mondor disease (MD), from magnetic resonance imaging that was performed in the previous hospital. A physical examination revealed a small pinhole lesion at the distal part of our patient’s penis and a rigid cylindrical tube extending to the proximal side of the penis.

**Fig. 1:**
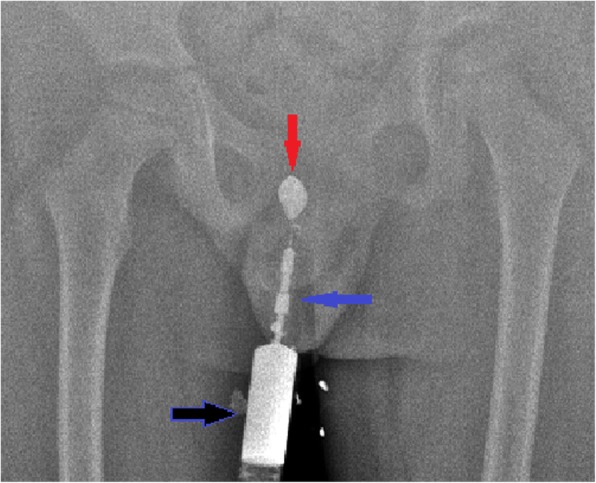
A sinusography performed using an injector (*black arrow*) showed that the sinus extended to the prepubic area (*red arrow*) and there was no passage of contrast material to the genitourinary system (*blue arrow* shows the tract of the congenital prepubic sinus)

**Fig. 2:**
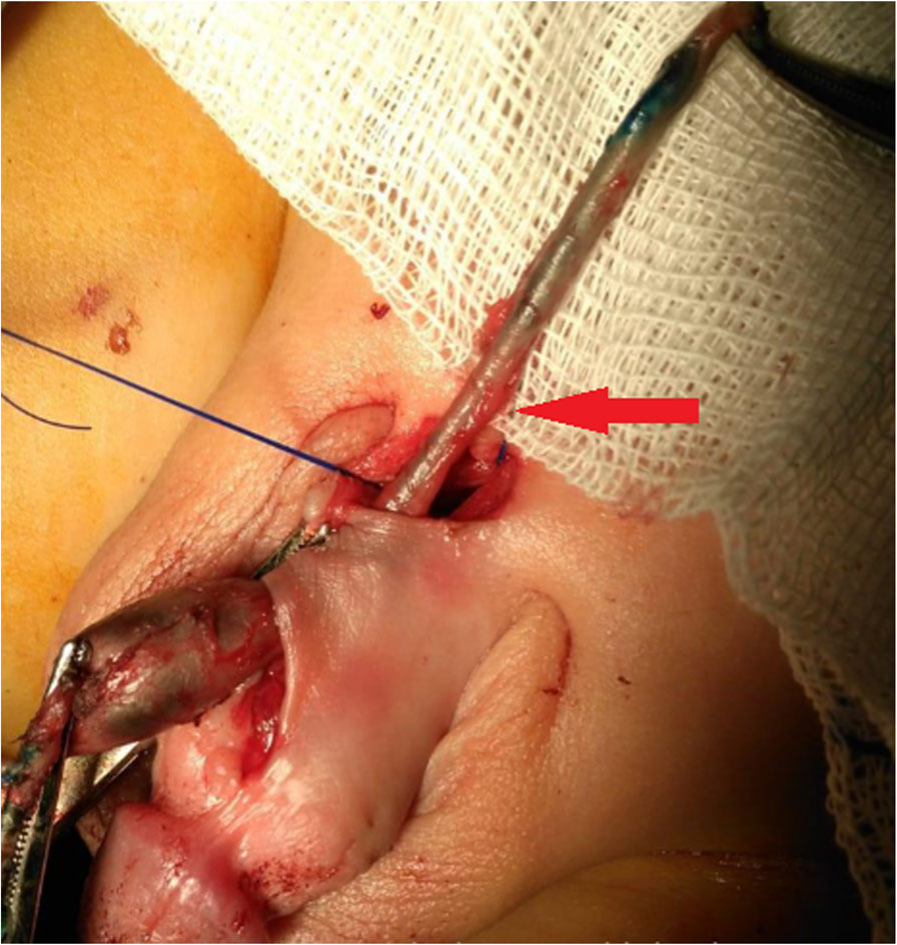
The length of the excised sinus and tract (*red arrow*) were 6 cm in length

**Fig. 3:**
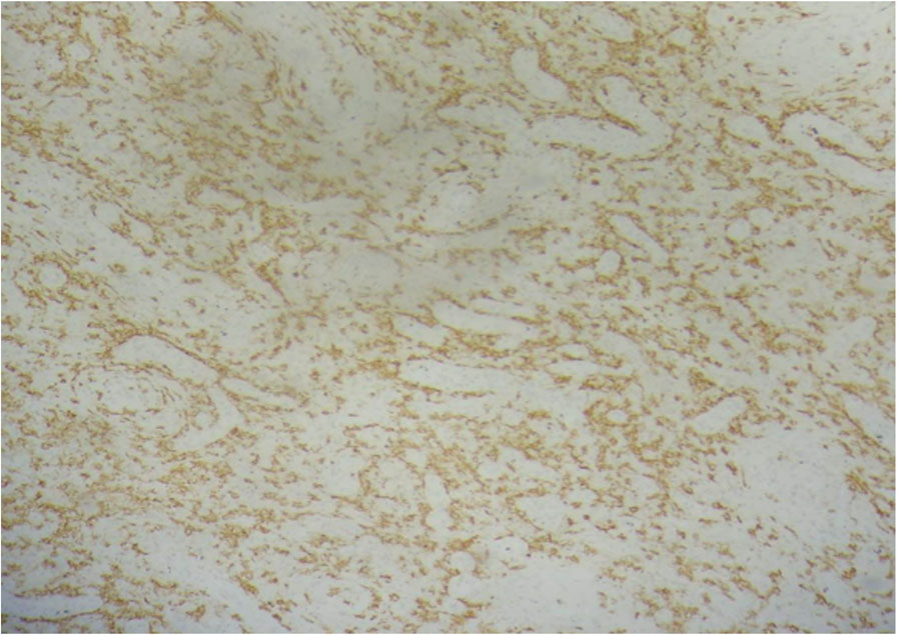
Intense chronic inflammation surrounding the squamous epithelium and the posterior support fibrous parenchyma

In laboratory analysis, his total white blood cell count was 6.1 × 10^3^/mm^3^, hemoglobin was 13.2 g/dL, alanine aminotransferase was 19 u/l, aspartate aminotransferase was 21 u/l, and creatinine was 0.5 mg/dl; serological tests were negative: hepatitis B surface antigen (HbsAg), anti-hepatitis C virus (HCV), and anti-HIV. Urine analysis showed normal amounts of red cells with suspicion of urinary tract infection. Due to the fact that he was treated with various antibiotics regimens, no bacterial growth was detected in the swab culture samples, which were obtained from the fistula mouth. Genitourinary system ultrasonography revealed no additional anomaly. Fistulography/sinography revealed that there was no relationship between his urinary tract and the sinus (Fig. 1). For treatment, surgical exploration was performed and a long sinus, apparently ending as a fibrous tract at the anterior surface of his pubic symphysis was found and resected (Fig. 2). No complications were observed after the operation and during 9-month follow-up period. Histological examination revealed that the inner mantle was a multiple lamellar squamous epithelium and intense chronic inflammatory infiltration was observed in the parenchyma of the fibrous support forming the wall, often associated with cyst epithelium (Fig. 3).

## Discussion

In this case presentation, we have presented a case of a pediatric patient with CPS presenting with purulent discharge following circumcision surgery. We have discussed the details of diagnosis and management of this rare and unusual entity.

CPS is a tract located at the skin overlying the symphysis pubis, superior to the base of the penis or clitoris, and extending proximally, but does not communicate with the anterior bladder wall [2, 3]. After the first report of CPS in 1987, around 50 cases have been reported in the literature so far [4]. Eroglu *et al.* reported an incidental diagnosis of CPS in a 7-year-old boy during circumcision. They stated that CPS can be overlooked due to its location between the skin folds and that fluid discharge or erythema at the suprapubic area should raise concern about CPS [5].

Although the etiology of CPS is not known exactly, four theories have been proposed to explain it [4]:

1. Anomaly of abdominal wall closure
2. A variant of dorsal urethral duplication
3. A fistula of primitive urogenital sinus
4. A remnant of primitive cloaca

Our histopathological results suggest that CPS may result from a cloacal defect or abdominal midline closure defect. Rozanski *et al.* proposed that CPS is a mild form of a midline abdominal wall closure defect [6]. The presence of stratified squamous epithelium in the entire tract might support the theory that it is an anterior abdominal wall closure. In a recently published study, Nazir *et al.* mentioned that CPS is an aborted dorsal urethral duplication or a cloacal remnant [4]. Soares-Oliveira *et al*. stated that CPS is a congenital fistula of the primitive urogenital sinus, with three anatomic subtypes depending on the direction of the sinus tract: high, toward the urachal remnant; middle, toward the bladder; and low, toward the prostatic urethra [7]. Tsukamoto *et al*. proposed that CPS may be caused by a residual cloacal membrane and umbilicophallic groove, and that the depth may determine the position of the end of the sinus tract [8]. Accordingly, with several different theories, the etiology of CPS still remains unclear.

The physical examination findings of our case mimicked the findings of superficial dorsal venous thrombosis, known as MD of the penis. In addition, it was evaluated as MD in magnetic resonance imaging, which was performed in the previous hospital. The patients who have MD usually admit with pain, and palpable swelling and hardness along the course of the dorsal vein. Thrombosed dorsal vein of the penis often extends to the suprapubic region, with associated erythema and edema of the penile skin. In our case, the finding of “rigid cylindrical tube extending to the proximal side of the penis” on physical examination brings MD to mind. However, the presence of “small pinhole lesion” and “purulent discharge” were not specific findings for MD. To differentiate between possible causes, we performed fistulography by injecting contrast material through a small angiocatheter and confirmed the diagnosis of prepubic sinus.

CPS is usually asymptomatic in neonates, and a diagnosis is often achieved later in life, after spontaneous extrusion of purulent material from the pre-existing hole in the pubic region, close to the root of the penis or clitoris. However, in our case, there was no fistula opening and purulent discharge began after circumcision. We could not find any publication in the literature stating a similar presentation: purulent discharge from the prominence following circumcision. In contrast to the cases in the literature, the opening of the distal fistula in our case was close to the coronal sulcus of the penis [7]. This condition suggests that inflammation after circumcision may have accelerated the fistula formation and extrusion of purulent material. However, infective complications of circumcision should be considered in the differential diagnosis. The presence of fibrous tract and sinus in the fistulography imaging eliminated the possibility of infective complications caused by circumcision. Our patient’s presentation and the clinical findings were features that distinguished our case from other cases in the literature.

In the treatment of CPS, complete excision of the tract is required to prevent recurring symptoms, infections, and potentially late malignant changes [9].

## Conclusions

Our case showed that penile MD and circumcision complications should be considered in the differential diagnosis of CPS. So, before CPS surgery, it is of critical importance to rule out penile MD and the possibility of circumcision complications (especially infective complications) mimicking CPS. Detailed anamnesis, careful physical examination, and specific imaging (for example, fistulography) can help to differentiate between various pathologies and to confirm the diagnosis of CPS.

## Abbreviations

CPS: Congenital prepubic sinus
MD: Mondor disease
